# Myositis ossificans occupying the thenar region: a case report

**DOI:** 10.1186/s13256-015-0586-8

**Published:** 2015-05-06

**Authors:** Tsutomu Akahane, Naoya Mori, Yukio Nakatsuchi

**Affiliations:** 1 Department of Orthopaedic Surgery, Shinshu-Ueda Medical Center, 1-27-21 Midorigaoka, Ueda, Nagano 386-8610 Japan; 2 Department of Orthopaedic Surgery, Marunouchi Hospital, 1-7-45 Nagisa, Matsumoto, Nagano 390-8601 Japan

**Keywords:** Hand, Myositis ossificans, Soft tissue calcification, Thenar region

## Abstract

**Introduction:**

Myositis ossificans is a benign, self-limiting, tumor-like lesion that usually affects the elbow and thigh; occurrence in the hand is uncommon. We report a rare case of a patient with myositis ossificans in the thenar region.

**Case presentation:**

A 15-year-old Japanese girl presented to our hospital with a 2-month history of a painful mass in the right thenar region without previous trauma. The clinical and radiological examination findings suggested an osteoblastic malignancy. A diagnosis of myositis ossificans was made on the basis of an incisional biopsy. Despite the location of the lesion in the thenar region, a normal functional outcome was achieved after marginal resection of the mature lesion.

**Conclusions:**

Clinicians should consider myositis ossificans as a possible diagnosis for a soft tissue mass in the hand, thereby avoiding unnecessarily aggressive therapy.

## Introduction

Myositis ossificans is a benign, tumor-like lesion of heterotopic ossification that affects the muscles, particularly those around the elbow and thigh; occurrence in the hand is uncommon [1]. Despite its being a clinically and histologically distinct entity, diagnosis may be considerably difficult, especially when it is located in an uncommon site. We report a rare case of a patient with myositis ossificans in the thenar muscles and discuss the differential diagnostic and surgical interventions performed.

## Case presentation

A 15-year-old Japanese girl was referred to our hospital because of a 2-month history of a painful mass in the dominant right thenar region. She had no history of acute injury and had not participated in any sports activity. An examination of the thenar region revealed that it was swollen and well circumscribed, and a firm mass was palpable. Minimal local tenderness and warmth were noted. Her thumb movement was slightly restricted, with pain. Her laboratory findings, including white blood cell count, erythrocyte sedimentation rate and C-reactive protein level, were normal.

**Figure 1:**
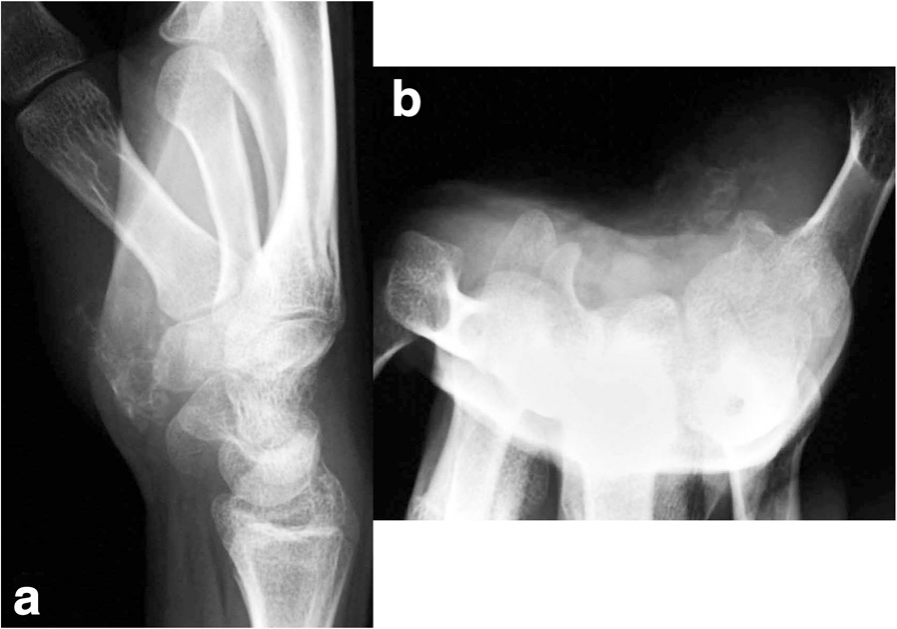
Radiographs of the case. **(a)** Lateral and **(b)** carpal tunnel aspects of the right hand showing ossification of soft tissues over the volar aspect of the thenar region.

**Figure 2:**
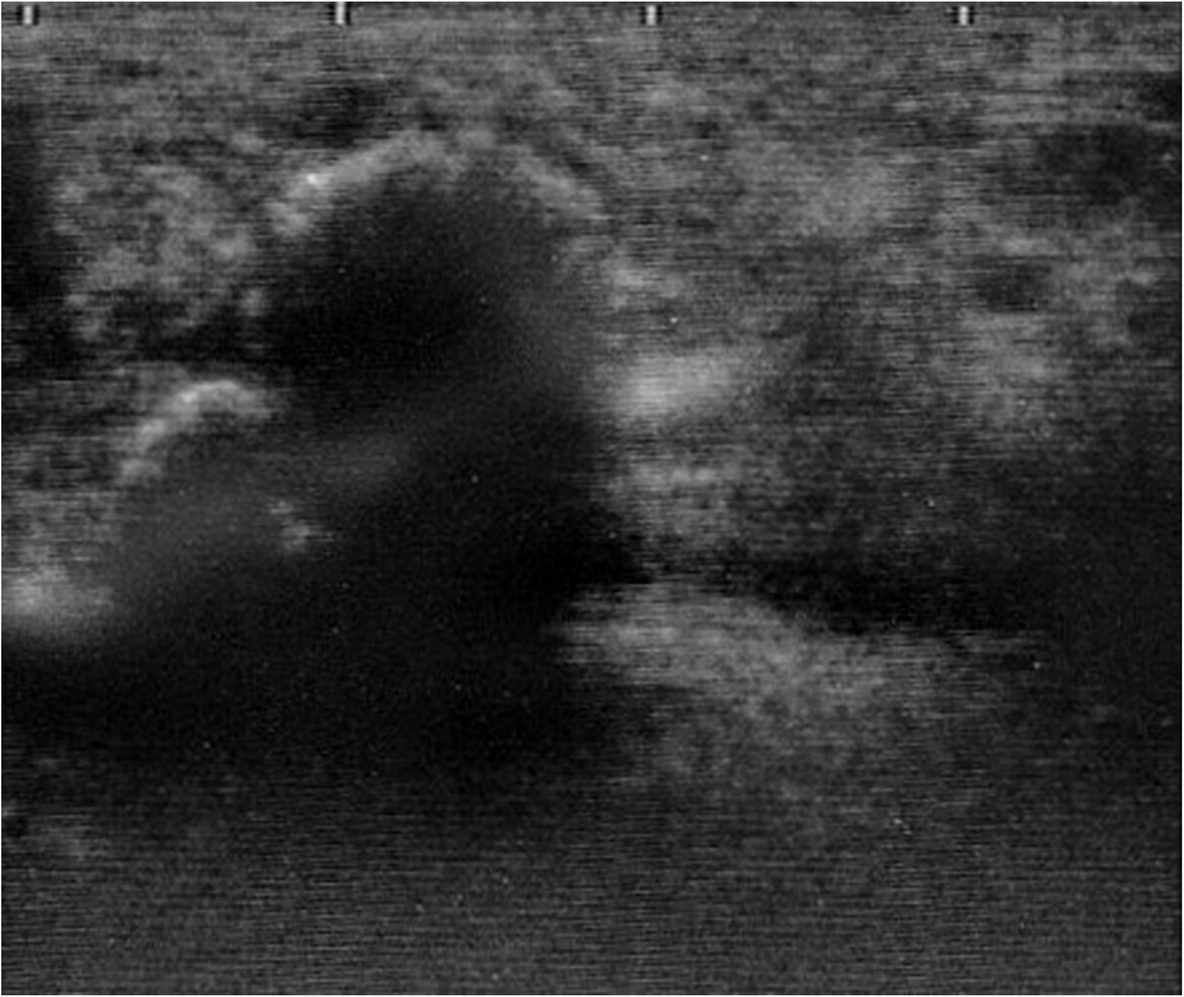
Ultrasound image of the hand. A lesion with a firm surface was present.

**Figure 3:**
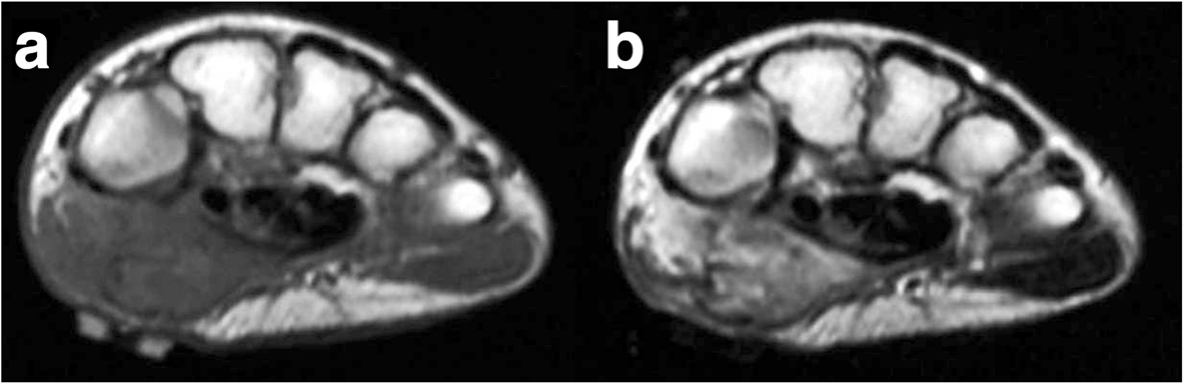
Axial magnetic resonance images of the hand. **(a)** T1-weighted (repetition time (TR), 561ms; echo time (TE), 20ms) and **(b)** T2-weighted (TR, 3500ms; TE, 90ms) images of the right hand showing a well-circumscribed mass occupying the thenar muscles.

Radiographs of the hand (Figure 1) showed well-demarcated calcification or ossification just volar to the carpals; there was no suspicion of a previous bone injury or periosteal reaction. Ultrasound (Figure 2) revealed a lesion with a firm surface. Magnetic resonance imaging (MRI) (Figure 3) showed a mass within the thenar eminence with peripheral edema.

**Figure 4:**
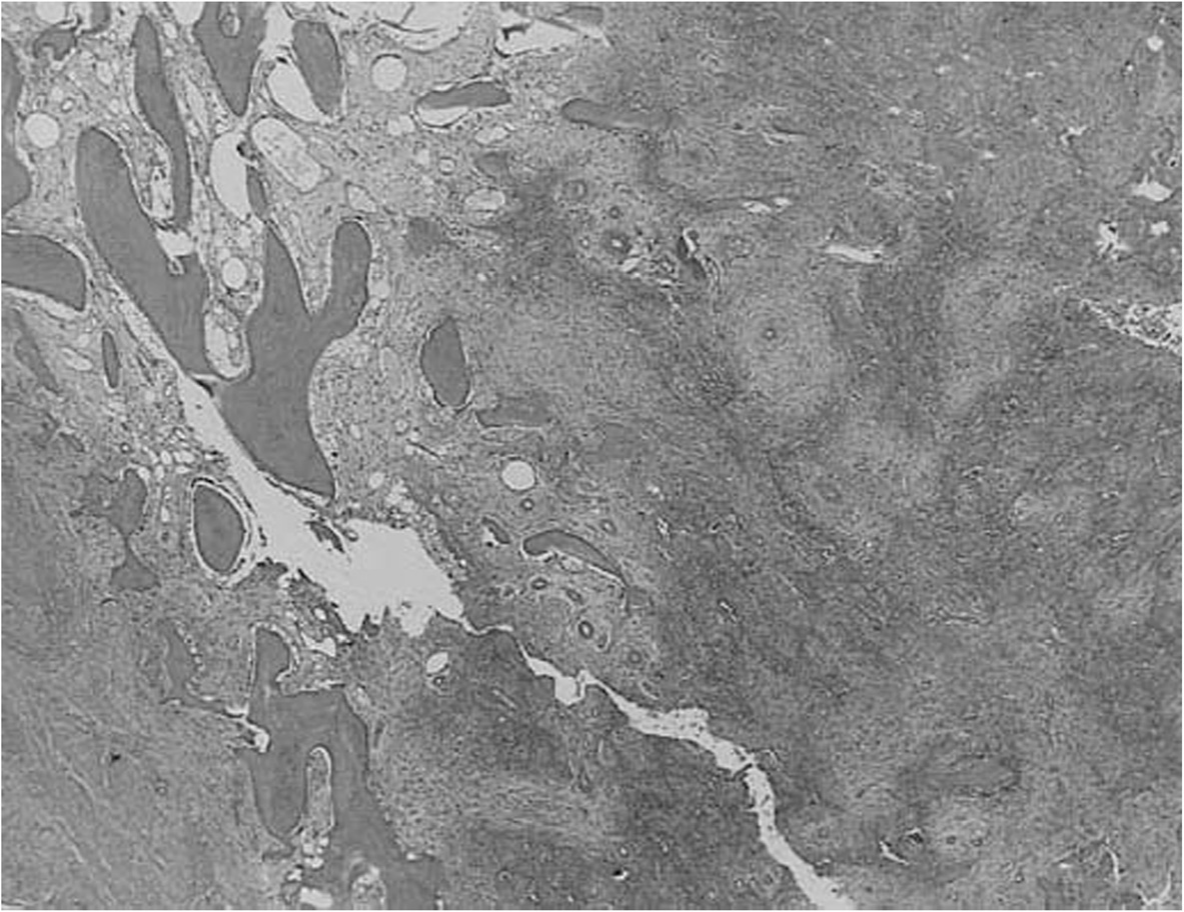
Biopsy specimen. Immature fibroblastic tissue with a mild degree of mitotic activity and some osteoid were observed. Hematoxylin and eosin staining; original magnification, ×10.

An incisional biopsy was performed for a definitive diagnosis. Histopathologically, most of the mass comprised immature fibroblastic tissue with a mild degree of mitotic activity, and some osteoid was also observed (Figure 4). The combination of histopathological and radiological findings revealed a distinct “zonal pattern”: The innermost portion of the mass comprised fibroblastic tissue; the intermediate zone comprised ill-defined trabeculae and/or osteoid; and the periphery comprised calcification and mature lamellar bone. On the basis of these histopathological findings, a diagnosis of myositis ossificans was made.

The patient’s pain persisted and occurred spontaneously, and the prognosis of the firm mass was unfavorable. We decided to perform a marginal resection after radiographically confirming the maturation of the lesion without accompanying destructive growth. Radiography showed that the superficial thenar muscles (the flexor pollicis brevis and its superficial head as well as the abductor pollicis brevis) remained intact; however, deeper structures such as the opponens pollicis and the deep head of the flexor pollicis brevis were completely occupied by the myositis ossificans. Both the adductor pollicis muscle and the flexor pollicis longus tendon were clearly separate from the lesion. The resected specimen was well circumscribed and showed a distinct zonal pattern of myositis ossificans characterized by the presence of fibroblastic tissue with chronic inflammatory cells, osteoid, mature lamellar bone and surrounding muscle.

The patient was treated with a splint post-operatively. At the 7-month follow-up examination, the patient was asymptomatic and exhibited normal function and a full range of motion of all digits of the hand, particularly thumb flexion, abduction and apposition. The surgical procedure produced a good outcome, as evaluated with the disability/symptom score of the Japanese Society for Surgery of the Hand version of the Disability of the Arm, Shoulder, and Hand questionnaire [2]; that is, 52.5 points pre-operatively and 7.8 points at follow-up.

## Discussion

Myositis ossificans is a localized, self-limiting, ossifying process that usually follows mechanical trauma: 60% to 75% of all cases involve trauma [1,3]. Identical lesions also occur in persons with no apparent history of preceding injury, and in some of these cases, an infectious process has been considered to be a possible cause or the initiating factor. Although our patient had no history of significant injury, whether minor repetitive injuries were present could not be ascertained.

Myositis ossificans involves the limbs in about 80% of cases. The most commonly affected sites in the lower extremity are the quadriceps and gluteus muscles, and those in the upper extremity are the flexor muscles, particularly the brachialis muscle [1]. However, involvement of the hand is rare, and only a few cases have been reported to date [4-8].

Myositis ossificans represents diffuse soft tissue swelling following injury. This swelling gradually develops into a firm mass [1], the periphery of which is the most densely calcified (zoning phenomenon) visualized by radiography or computed tomography. The lesion has also been shown to be a heterogeneous mass producing extensive muscle edema on MRI scans [9].

When myositis ossificans occurs in its common locations and/or if a clear clinical history and findings are obtained, the diagnosis is not complicated. However, the differential diagnosis may be a problem in patients with a lesion that lacks the characteristic zoning phenomenon and grows in an irregular multifocal or multilobulated fashion [1]. In our patient, the rare location of the lesion and the lack of previous injuries necessitated histopathological assessment to distinguish the lesion from extraskeletal osteosarcoma. In contrast to myositis ossificans, osteosarcoma presents a more disorderly growth of hyperchromatic and pleomorphic cells with osteoid formation. Additionally, the greater degree of cellular atypism and infiltration of neighboring tissues in a destructive manner are highly indicative of this lesion. Although mitotic figures are present in immature myositis ossificans and osteosarcoma lesions, clearly atypical or tripolar forms point toward malignancy [1].

Because myositis ossificans is a benign, self-limiting process, its prognosis is excellent. There is no need for further therapy once the diagnosis of myositis ossificans has been established by biopsy or local excision [1]. However, aggressive surgical treatment may be required to alleviate intractable pain due to compression of neural tissue and limitation of function [5]. In our patient, we decided to perform surgery after the lesion matured. Intra-operatively, we found that the myositis ossificans occupied the vast majority of the thenar region, particularly the opponens pollicis and the deep head of the flexor pollicis brevis. The patient’s post-operative course was nevertheless uneventful, and no functional deficit was apparent.

The following thumb functions are coordinated by several muscles: Thumb flexion is integrated by the flexor pollicis longus and flexor pollicis brevis, extension by the extensor pollicis longus and extensor pollicis brevis, abduction by the abductor pollicis longus and abductor pollicis longus brevis, and adduction by the adductor pollicis and first dorsal interosseous. Thumb apposition is controlled by the coordinated movement of the abductor pollicis longus, abductor pollicis brevis, opponens pollicis and flexor pollicis brevis. The affected deep heads of the occupied opponens pollicis and flexor pollicis brevis were not extensively involved in the thumb discordance.

## Conclusions

We report a rare case of a patient with myositis ossificans that occupied the thenar muscles. Surgical intervention enabled normal function. Because of the rare location of the lesion, incisional biopsy was necessary to establish the diagnosis.

## Consent

Written informed consent was obtained from the patient’s legal guardians for publication of this case report and any accompanying images. A copy of the written consent is available for review by the Editor-in-Chief of this journal.

## Abbreviations

MRI: Magnetic resonance imaging
TE: Echo time
TR: Repetition time

## References

[1] Weiss SW, Goldblum JR. Osseous soft tissue tumors. In: Enzinger and Weiss’s soft tissue tumors. 4th ed. St Louis, MO: Mosby; 2001. p. 1389–405.

[2] Imaeda T, Toh S, Nakao Y, Nishida J, Hirata H, Ijichi M, et al. Validation of the Japanese Society for Surgery of the Hand version of the Disability of the Arm, Shoulder, and Hand questionnaire. J Orthop Sci. 2005;10:353–9. A published erratum appears in J Orthop Sci. 2007;12:107. 10.1007/s00776-005-0917-5 PMC2780667 16075166

[3] Parikh J, Hyare H, Saifuddin A. The imaging features of post-traumatic myositis ossificans, with emphasis on MRI. Clin Radiol. 2002;57:1058–66. 10.1053/crad.2002.1120 12475528

[4] Schütte HE, van der Heul RO. Pseudomalignant, nonneoplastic osseous soft-tissue tumors of the hand and foot. Radiology. 1990;176:149–53. 10.1148/radiology.176.1.2112767 2112767

[5] Kusuma S, Lourie GM, Lins RE. Myositis ossificans of the hand. J Hand Surg Br. 1999;24:128–30. 10.1054/jhsb.1998.0046 10190626

[6] Jayasekera N, Joshy S, Newman-Sanders A. Myositis ossificans traumatica of the thenar region. J Hand Surg Br. 2005;30:507–8. 10.1016/j.jhsb.2005.06.011 16084631

[7] De Smet L, Degreef I. Myositis ossificans of the hand in a child: case report. J Pediatr Orthop B. 2012;21:539–41. 10.1097/BPB.0b013e3283524bfa 22568961

[8] Chadha M, Agarwal A. Myositis ossificans traumatica of the hand. Can J Surg. 2007;50:E21–2. PMC2386223 18067695

[9] Ehara S, Nakasato T, Tamakawa Y, Yamataka H, Murakami H, Abe M. MRI of myositis ossificans circumscripta. Clin Imaging. 1991;15:130–4. 10.1016/0899-7071(91)90162-o 1913313

